# Sleep Deprivation Can Lead to Increased Physiologically Based Irritability in College Students

**DOI:** 10.5964/ejop.17555

**Published:** 2026-05-29

**Authors:** Jaka Prevodnik, S. Elizabeth Rácz-Brunner

**Affiliations:** 1 Chemistry, Biology, and Health Sciences Department, South Dakota School of Mines and Technology, Rapid City, SD, USA; Università Cattolica del Sacro Cuore, Milan, Italy

**Keywords:** irritability, sleep deprivation, mood, college students, sleep

## Abstract

**Objectives:**

While sleep deprivation has been thoroughly researched in association to cognitive and physiological effects, very few have evaluated its association with irritability. This study addresses the relationship between sleep and irritability using physiological data and includes a research model to aid future experiments on irritability and neighboring mood concepts.

**Methods:**

Thirty-four US undergraduate students participated in a physiologically based study on irritability. Participants were separated into two groups based on sleep recommendations adapted from the Centers for Disease Control and Prevention (CDC) guidelines for college students. The experiment consisted of three cognitive tasks paired with irritable stimuli, all done while undergoing heart rate (HR) measurements.

**Results:**

The mean length of time participants slept throughout the study was 7.70 hours, which was higher than initially expected. Significant increases seen in HR during periods of the Insoluble Puzzle task possibly indicate that task duration, perceived reward blocking, and participant expectation can play a significant role in inducing irritability when sleep deprived (*p* = .014, *p* = .045).

**Conclusions:**

These findings indicate that exposure to an irritating stimulus can have a physiological impact on a sleep-deprived individual. Importantly, this study is also practically applicable to other researchers as it presents a research model that serves as a guideline for future research on irritability and its neighboring concepts.

Sleep deprivation is a prevalent problem among college students and its effects on academic performance, mood, and cognitive functioning are well-known (Alhola & Polo-Kantola, 2007; Chattu et al., 2018; Patrick et al., 2017; Short & Louca 2015; Thompson et al., 2022). The main factors that contribute to loss of sleep time among college students are caffeinated and alcoholic drink consumption, use of stimulants, and screen-based technology before bedtime (Hershner & Chervin, 2014). The recommended duration of sleep for college-aged students is a minimum of 7 hours per night (CDC, 2022). However, cognitive and behavioral deficits can arise with the restriction of an individual’s optimal time in bed (Banks & Dinges, 2007). While the effects of sleep deprivation on mood changes and stress have been investigated and supported (Liu et al., 2020; Minkel et al., 2012; Nollet et al., 2020; Saghir et al., 2018; Thompson et al., 2022), its effect on irritability has mainly remained unclear (Whiting et al., 2023).

Irritability is a mood distinct from anger and aggression (Gröndal et al., 2023; Toohey, 2020; Toohey & DiGiuseppe, 2017; Vidal-Ribas et al., 2016). However, most psychological definitions of irritability overlap with anger and aggression in the DSM-5 from 2013 (Toohey & DiGiuseppe, 2017). Recent definitions of irritability talk about it as a state of higher sensitivity to sensory stimuli, which results in a higher probability of responding to the stimuli with anger or aggression (Gröndal et al., 2023; Toohey & DiGiuseppe, 2017). According to an international study conducted in ten different countries across five continents, irritability was also found to be an independent and universal mood (Toohey, 2020). Additionally, the study revealed that almost half of the participants thought that anger and irritability were the same or directly related. This prevalent miscategorization of irritability is a major obstacle when it comes to properly defining and measuring it (Barata et al., 2016; Bell et al., 2021; Gröndal et al., 2023; Saatchi, Agbayani et al., 2023; Toohey & DiGiuseppe, 2017). Several attempts have been made to clearly define the construct of irritability (Gröndal et al., 2023; Saatchi, Olshansky, Fortier, 2023; Toohey, 2020; Toohey & DiGiuseppe, 2017). To date, Toohey’s and DiGiuseppe’s (2017) review of irritability provides the most comprehensive conceptualization of irritability.

To experimentally study irritability it is necessary to provide a clear definition and determine and account for any manner in which its expression differs from anger and aggression. Detailed definitions of irritability most commonly refer to it as a state, where a non-specific cue could more easily and strongly provoke an individual in a way that would be considered out of proportion for such a stimulus (Deveney et al., 2019; Toohey & DiGiuseppe, 2017). Additionally, irritability is a reactive physiological state, directly linked to changes in physiological or biological processes, and is not necessarily outwardly expressed (Gröndal et al., 2023; Saatchi, Olshansky, Fortier, 2023; Toohey & DiGiuseppe, 2017). These characteristics commonly manifest in situations where a goal is blocked (frustrative non-reward) or when physiological needs are not met, as the reactions are less likely to be inhibited (Saatchi, Olshansky, Fortier, 2023; Toohey & DiGiuseppe, 2017). Since irritability can predispose anger and aggression, it also has a link with frustration. According to the reformulated Frustration-Aggression Theory by Breuer and Elson (2017), irritability is one of the mediating factors between negative affect and aggressive inclinations. Its link to frustration, if considered bidirectional, means irritability could be the cause for frustration or could be provoked by frustration (Barata et al., 2016; Saatchi, Olshansky, Fortier, 2023).

Since obtaining an independent definition in the mid-2010s and becoming established as a non-specific indicator for some affective and mood disorders, irritability has garnered more interest as a potential diagnostic tool in psychiatry (Saatchi, Olshansky, Fortier, 2023; Vidal-Ribas et al., 2016). Studies have shown that irritability, in addition to agitation and anxiety, can be used to identify patients with depressive symptoms, which means it may have psychiatric diagnostic value (McIntyre & Weiller, 2015). The primary difficulty when studying irritability is that irritability is not necessarily visibly expressed (Gröndal et al., 2023). This has led to self-reporting measurement tools as the main instrument employed to capture and study the state of irritability. Yet, a recent finding linked an increased heart rate (HR) compared to baseline, however, no mechanisms for cardiovascular arousal were found (Naim et al., 2021). A link between HR and irritability is consistent with the current definition of irritability, a state directly linked to changes in physiology (Toohey & DiGiuseppe, 2017).

To date, only one study has previously directly tested the association between sleep deprivation and irritability in healthy individuals; it used the self-reported measurement approach (Whiting et al., 2023). The Whiting study found a likely link between irritability and sleep deprivation (*p* < .001), but no direction of the association could be definitively determined due to the nature of the study design, as participants self-reported irritability via questionnaire.

The aim of the present study, after establishing the existence of a link between irritability and sleep deprivation in college students aged 18–21, was to investigate the directional relationship between sleep and irritability. Irritability was measured physiologically — via heart rate. Shorter than recommended sleep duration was expected to contribute to increased levels of irritability measured by HR. Since there are other physiological conditions associated with HR changes, such as stress or anxiety, a mean individual HR rate was calculated for each 30-second period. To aid in the goal of the present study a research model was developed. This was extremely valuable in ensuring the multiple aspects of our experiment, the physiological measurements from three separate cognitive tasks and rest periods, planned background noise, and participant-reported components came together precisely and were communicated efficiently and effectively.

## Method

### Study Participants, Recruitment, and Design

From August to October of 2023, 34 participants (70% male; *M* = 18.9 years, *SD* = 0.9 years) took part in this study on sleep deprivation and irritability conducted at South Dakota School of Mines and Technology (SD Mines). Recruitment occurred exclusively on the SD Mines campus. Participants were recruited in person, via flyers on campus, and by word of mouth. Participants were informed that the study involved cognitive function testing as it relates to sleep patterns. No information was provided regarding the true goal of the study until the end of the experiment. Participants were requested to refrain from speaking to others about the experiment post-participation to prevent giving hints to incoming naïve participants. Exclusion criteria included persons under 18 or over 25 years old and anyone not a full-time student at SD Mines. All identifying information on participants was anonymized. All participants provided written informed consent, and the study was approved by South Dakota State University’s Institutional Review Board (6/14/2023, IRB-2306004-EXP).

**Figure 1:**
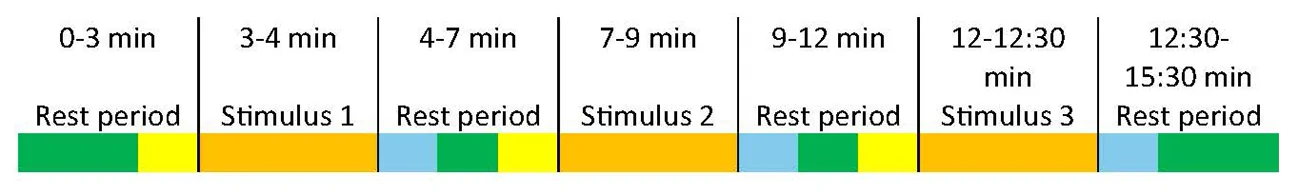
Timeline of Testing and HR Recording Procedure *Note.* Green = rest period, Yellow = pre-stimulus period of rest period, Orange = stimulus period, Blue = post-stimulus period of rest period.

Participants were assigned into groups based on their self-reported sleep diaries. The sleep diaries included data (see Rácz-Brunner & Prevodnik, 2025) for a minimum of five consecutive days and up to seven days prior to testing. Sleep deprivation was defined as sleeping 7 hours or less on average, adapted from the Center for Disease Control and Prevention (CDC) guidelines. Exactly seven hours was included in Group 2 calculations due to sleep diary rounding error. For the purposes of data analysis, participants with an average sleep duration of over seven hours were assigned into Group 1 and those with seven hours or less were assigned to Group 2. Missing any days in the sleep diary would result in exclusion from the study. For all study treatments and tasks, both groups underwent the same procedures. Testing occurred between 12:00 and 17:00 over four weeks between September and October. Measurements were taken before, during, and after each task (see Figure 1). Additional demographics were collected on participants prior to participation, i.e., age and biological gender.

### Cognitive Tasks and Irritability

The first task (T1) was a standard Stroop Color and Word Test which induced a cognitive workload and was paired with implicit expectation and benign background noise. While participants were performing the task, researchers spoke in low voices according to a pre-planned script to simulate a study environment in a college library. Additionally, a note with an extremely above-average score was subtly placed by the participant’s workspace for them to look at in passing. The second task (T2) was the Insoluble Puzzle, inspired by Feather (1961), which presented a clear expectation of its goal but made it impossible to reach. For T2, participants were instructed to match various plastic shapes to a pattern on a card. For the first puzzle, participants were given an easy puzzle to complete, consisting of three pieces. However, the second puzzle was unsolvable, and participants were given plausible but incorrect pieces to match the required shape. The third task (T3), the Unpredictable Quiz, although academically straightforward, was taken away prematurely without the chance to finish the quiz. Participants were told the quiz would end after two minutes, but it was physically taken away after 30 seconds.

Rest periods occurred before the first task, between tasks, and after the last task as a key part of the study design. The rest periods let the participant return to a baseline, whether it was after their initial arrival or completion of a task. Each rest period lasted three minutes. The last minute before any stimulus was used as a pre-stimulus HR reading, and the first minute after any stimulus was used as a post-stimulus HR recovery reading.

### Physical Measurements

Heart rate (Polar(R) Heart Monitor) was measured using the iWorx™ device. The HR monitor was strapped around each participant’s chest in the appropriate location and in contact with the skin. The HR monitor was cleaned per the manufacturer’s instructions between each participant. Measurements and notes were taken during all three stimuli and four rest periods.

### Statistical Analyses

General summary statistics were conducted. Participants were compared to themselves first with a “pre-stimulus and during-stimulus” average, and a “pre-stimulus and post-stimulus” average. Then, participants were split into groups based on their average sleep duration. Unpaired Welch’s *t*-tests, one and two-tailed, were performed on the two groups as appropriate after unequal variance was confirmed between groups.

### Research Model

To optimally conceptualize, construct, reassess, and refine our study, we developed a research model. Specifically, a research model that better supports the complete image of the test as experienced by the subject, and which aspects of the study researchers want to focus on and how. This allows 1) researchers to formulate accurate images of the test repeatedly, 2) groups of researchers to conceptualize testing for analysis as a group better (especially if not all researchers were present at testing), 3) the establishment of a clear mode of communication that researchers can use throughout the field, and 4) is highly flexible and easily modifiable.

The framework of the research model, based on our study design, allowed for flexibility in adding design components. The flexibility of the research model contributed to the empirical rigor of the study by reducing error and providing a foundation on which the researchers could conceptualize testing, reliably track results for comparability, and pivot their actions in response. The research model easily changed with the needs of the researchers as the study progressed. Once laid out, the main table helped clarify points and eventually informed back on our study design, as shown in Table 1. We started our main table by identifying the tests we planned to conduct. Then, we grouped the various aspects of those tests of interest into discrete categories, see methods below. As research progressed, tests, categories, and details of the framework were edited and refined as needed.

**Table 1:** Research Model Components

Factor	T1	T2	T3
Expectation	1, S	1, D	0
Distraction	1, I, S	0	0
Timed	1, S	1, D	1, S
Measure	1, P	1, P	1, P
Duration	S	L	S
Stimulus type	V, K	S, K	V
Results	0, 1	1, 1	0, 1

*Note.* T = test; Expectation: D = directly S = subtly; Distraction I = Intentional, S = Subtle; Timed: S = Subtle, D = Direct; Measure: P = Physical; Duration: S = Short, L = Long; Stimulus Type: V = Visual, K = Kinetic, S = Spatial; Results: 0 = not statistically significant, 1 = statistically significant at *p* < .05 level; Unless otherwise specified for all observations 0 = none, 1 = present.

Our research model successfully delineates detailed characteristics of our study, showing how effectively subtle and important nuances are captured and communicated, see Table 1. Specifics on each of seven characters comprising our research model (Expectation, Distraction, Timed, Duration, Measure, Stimulus Type, and Results) are included to add detailed structural insights for each test conducted.

1. **Expectation:** This includes whether or not the test has an expectation associated with it from the perspective of the test taker. For example, we found it important to track if an expectation was present, if it was communicated to the test taker before or during the test, and if an expectation was communicated was it done directly or subtly.
2. **Distraction:** Creating a benign dialogue read at a normal speaking level during a test to potentially distract subjects from testing, as we did in this study, is an example of an intentional, but subtle distraction. We included events that distracted a subject intentionally or inadvertently.
3. **Timed:** “Is the test timed?” was a straightforward starting point for this component of the model. All of our tests were timed. However, this component also indicates how the test was timed. Was the test time subtly marked with verbal instructions? Or was there a countdown clock that the participant watched throughout testing, i.e., was there subtle or direct added pressure from the subject’s perspective?
4. **Duration:** This section describes the duration of each test taken by study participants, including the order in which they took them. Herein, our participants spent 1 minute completing a Stroop test to the best of their ability (coded as “Short”), 2 minutes solving progressively harder (ultimately unsolvable) spatial puzzles (coded as “Long”), and 30 seconds answering straightforward questions on the Unpredictable Quiz — after subjects were told they would have 2 minutes (coded as “Short”), see Table 1.
5. **Measure:** A clear statement of which measurements (physical, survey, other) were conducted and to what they were tied. For example, we used a physical measurement of heart rate to capture participants’ status of irritability. We coded for testing intervals of relatively short duration (2 minutes) leading to the type of stress responses evidenced via monitoring heart rate (Naim et al., 2021).
6. **Stimulus Type:** The main stimulus type was designated based on a test’s principal task, e.g., visual, kinetic, or spatial. Other stimuli options or combinations are possible as appropriate. In this study, three tasks were conducted: one visual, the Stroop Test, the second was spatial, the Insoluble Puzzle, and finally, another visual, the Unpredictable Quiz, see Figure 1. The inherent nature of a stimulus type should be taken into consideration when devising the order of tests due to potential impacts on irritability. Directly defining the main stimulus type for each test provides all researchers, especially those not present at testing, an immediate snapshot of the focus of the test. Two of the tasks in our study also had a secondary stimulus (kinetic). This is listed secondarily in the model, see Table 1.
7. **Results:** Tests with statistically significant p-values are indicated here, i.e., *p*-values < .05. Observations on points relevant to the outcome of the study, interruptions, statistical test type, or instrumentation issues are also coded in this section, Present = 1, none = 0. For example, in this study on irritability there are notes on the subjects’ state of irritability versus anger and on self-soothing behaviors, see SI.

## Results

### Sleep Duration

The mean length of time participants slept throughout the study was *M* = 7.70 hours (*SD* = 0.67 hours). On average, Group 1 (*n* = 27) slept *M* = 7.95 hours (*SD* = 0.50 hours), while Group 2 (*n* = 7) slept *M* = 6.75 hours (*SD* = 0.23 hours). The overall mean sleep length of the sample was above the recommended sleep duration per CDC guidelines. This was due to a high percentage of participants (79%) with a self-reported sleep duration slightly above 7 hours. More than half (55%) of the participants fully or predominantly rounded their nightly sleep total to the half or full hour.

### Heart Rate Evaluation

Mean HRs across thirty-one 30-second intervals were used for analysis. Each rest period accounted for six 30-second intervals (see Figure 1). Stimulus 1 accounted for two of the 30-second intervals, stimulus 2 for four, and stimulus 3 for one. The mean HR of each interval (rest period or stimulus) was calculated and compared for each individual. The difference between HRs in each interval was then compiled and compared between groups. A significant increase in HR was observed between “pre-stimulus 2” to “during stimulus 2” (*M* = 3.13, *t* = 2.60, *p* = .014). Additionally, a significant difference in HR occurred between “pre-stimulus 2” and “post-stimulus 2” (*M* = 3.07, *t* = 1.81, *p* = .045). Importantly, there was no significant difference found in HR between rest periods. No other significant differences were found between pre- and post-stimulus and pre- and during-stimulus HR for other stimuli.

### Task Performance and Observations

Stimulus 2 was verbally reported as the most irritating task by half (50%) of the participants after the completion of the experiment. None of the participants reported feeling angry when completing the tasks (T1–T3). Only two participants exhibited an outward escalation in their expression (slapped the desk) upon learning the real purpose of the experiment at debriefing, possibly sourced from irritability.

### Research Model

The model provides an easy assessment of the number of tests, number and type of stimuli participants encountered per test, and in what sequence. The research model is a convenient stepping-off point for communication. Its abbreviated and flexible format leads to an efficient tailored environment and an organized workspace. On a fine scale (e.g., solo research, a single research study with/without language barriers or collaboration with a large research group with/without a multilingual component) characteristics and traits of each test are clarified to ensure there is a central framework accessible to all researchers, around which equitable communication can occur to form, execute, analyze, and complete the research project. Thereby increasing the likelihood of cultivating and catching insights from all researchers all while improving overall organization. On a larger scale (e.g., small, or large group meta-analyses with/without multilingual components) use of the research model leads to more accurate, structured, and effective meta-analyses. There is an important and unique opportunity to embrace a research model to strengthen how data across the subfield is organized—practically, for field-wide collaboration and ultimately for better results for all downstream applications from pure research to clinical practice.

## Discussion

This is the first study to physiologically test via HR whether sleep deprivation can influence the levels of irritability, as defined by Toohey and DiGiuseppe (2017), in college students. Crucially, pre- and post-stimulus physiological data comparisons are controlled for stress and anxiety, which are known to moderately correlate with irritability (Whiting et al., 2023). After controlling for those extraneous variables, sleep deprivation was found to significantly affect levels of irritability based on HR measurements during irritability-evoking stimuli. Our results were consistent with previous research studying the link between sleep and irritability (Naim et al., 2021; Whiting et al., 2023). Limitations inherent to the study design prevented a prior study, where irritability was self-reported via survey, from determining the directionality of the interaction between sleep and irritability (Whiting et al., 2023). Conversely, our experimental study was able to provide support for a directional relationship between these two concepts, as reflected in HR measurements. This was consistent with previous findings showing that sleep has a greater effect on mood than the effect mood has on sleep (Hickman et al., 2024; Triantafillou et al., 2019). Our findings show that the recovery to the resting HR was significantly slower in Group 2 than in Group 1 (*M* = 3.07, *t* = 1.81, *p* = .045, one-tailed), indicated by significantly increased HR levels between pre- and post-Stimulus 2 averages. Additionally, the task HR was significantly higher in Group 2 than in Group 1 (*M* = 3.13, *t* = 2.60, *p* = .014, two-tailed), which was captured by increased HR levels between pre- and during-Stimulus 2 HR means.

Unexpectedly, only values surrounding Stimulus 2 had statistically significant effects on irritability levels amongst participants (“pre-Stimulus 2” to “during Stimulus 2” *p* = .014; “pre-Stimulus 2” and “post-Stimulus 2” *p* = .045), as there were no significant HR differences found between groups in Stimulus 1 and Stimulus 3. This is possibly due to the fact that Stimulus 2 was the longest task in the experiment. It ran 1 minute and 1.5 minutes longer than Stimulus 1 and Stimulus 3, respectively. Additionally, reward blocking and expectation were likely the most evident in Stimulus 2 compared to the other two stimuli. The rest of the puzzles were always in the participant's visual field and perceived low difficulty made the reward blocking the most apparent. Furthermore, Stimulus 2 was the only stimulus that involved timed reminders by the experimenters of how much time remained, e.g., 1min, 30s, 15s, 10s, 5s, 4s, 3s, 2s, 1s, done. This possibly put more pressure on participants to solve the puzzle, likely affecting the sleep-deprived Group 2 more than Group 1. Future studies should focus on how varying duration affects sleep-deprived individuals and at what point the duration of reward blocking leads to a significant difference in irritability levels. In addition, duration should be further studied with the timing of a stimulus and how that could increase the perception of reward blocking and irritability levels.

Experimentally, studying the concept of irritability presents unique challenges. It is essential to ensure appropriate parameters for several key aspects of experimentation, specifically test type, timing, duration, and the extent of reward blocking. Too little reward blocking, a short test duration, and infrequent timing of a task could fail to evoke irritability. On the other hand, too much reward blocking, too long of a test duration, and constant reminders of how much time remains for the test could result in too much frustration and irritability — possibly leading a participant to progress to anger and/ or aggression. Such a progression may lead to a potential catharsis (Breuer & Elson, 2017), the order of the stimuli and the severity of each stimulus characteristic needs to be carefully considered prior to conducting any kind of testing. In this study, no participant outwardly expressed strong emotions during the experiment. All participants, except for one — who spontaneously guessed — were unaware of why they could not solve the Stimulus 2 puzzle. However, two participants experienced catharsis (slapped the desk) during debriefing when it was revealed that Puzzle 2 in Stimulus 2 was impossible to solve. Additionally, when asked for “any further comments or questions” none of the participants reported feeling angry during testing. Future studies could push the limit to determine which characteristics can evoke anger in order to detect any differences between different populations when it comes to the mediation of negative moods and aggressive inclinations. There is a need to evoke and measure irritability experimentally. Attempting to capture an accurate measurement of irritability based on self-reported questionnaires alone is highly subjective (Gröndal et al., 2023; Toohey & DiGiuseppe, 2017). Unless the potentially high levels of subjectivity in participant responses regarding irritability are adequately resolved, the use of questionnaires alone in scientific studies falls short of effectively measuring irritability.

Chronic stress and anxiety were controlled for as extraneous variables by the use of 3-minute rest periods, 1-minute acclimatization period, and non-invasive HR measuring methods. A period of 3-minutes was sufficiently long enough to allow HR to return to rest HR levels, while short enough to maintain participant engagement. Importantly, after comparing group means between all four rest periods no significant HR base-rate differences were found. This was as expected when chronic stress and anxiety are effectively eliminated as significant extraneous factors. In addition, the 1-minute acclimatization period effectively allowed participants to settle in, familiarize themselves with the environment, and ask questions regarding the physical measurements. The study used a simplified HR measurement device, the Polar(R) Heart Monitor instrumentation, which was easily, privately belted around the thoracic cage. Preventing any additional stress from cumbersome instrumentation, i.e., an EKG with five electrodes.

### Limitations

A limitation of this study was that it was conducted at a rural, STEM-focused college, the sample reflected campus gender composition, which was predominantly male. Also, due to the sample size of the study and convenience sampling methodology, its data should be generalized cautiously. However, using a design where HR periods were first compared within an individual, thus becoming their own control, increased our confidence in the validity of the experimental outcomes. Furthermore, statistically non-significant differences between rest periods provided additional evidence that confounding variables were controlled adequately, therefore increasing experimental validity. To build on this study’s results, future experiments should focus on recruiting a larger, more random, and more diverse sample. Finally, this study used a sleep diary to track participants’ sleep, which, much like questionnaires, is self-reported. To improve upon this common practice, a more objective method could be used, such as the use of actigraphy.

### Conclusion

This study provided first physiological evidence for the causal relationship between sleep deprivation and irritability. For a long time, the main difficulty studying irritability has been its definition and overlap with neighboring concepts. A recent systematic review by Toohey and DiGiuseppe (2017), brought more clarity to the field and opened the door to experimental research on irritability. To account for specific characteristics of irritability in our study and to prevent measuring neighboring concepts, a research model was constructed.

Our findings are mainly relevant to a college student population as this study is composed entirely of college students. Across all participants, mean sleep length was reported at 7.70 hours per night. It is likely that these numbers were overestimated by participants, due to self-reporting in the sleep diary (Clegg-Kraynok et al., 2023, Lauderdale et al., 2008). It is possible that participants wrote down the times they were in bed, not when they actually fell asleep. If most students are barely sleeping the recommended sleep time chronically, this could affect their irritability levels during the day and their reaction—depending on the length and reward blocking present in a situation. Future studies should focus on obtaining a larger more diverse sample, and use tools like a sleep ring, or other reliable monitoring devices that accurately measure exact quality and duration of sleep.

This small investigative study presents a first step in studying the concept of irritability experimentally without the need to exclusively use questionnaires. Our findings have significant implications for college students and indicate the potential consequences of their sleeping habits. It suggests that current sleep habits and deprivation may have social and learning impacts in situations where reward blocking occurs over a period of time. This study also has potentially significant relevance for future research on irritability and neighboring concepts by presenting the research model. This research model allows for efficient and effective comparison between studies and may be generally applied as a guideline in the development of research on irritability.

## Supplementary Materials

**Table:** 

Type of supplementary material	Availability/Access
Data	
Data 1	Rácz-Brunner and Prevodnik (2025)
HR Data 2	Rácz-Brunner and Prevodnik (2025)
Code	
No code provided	—
Material	
No study material provided	—
Study/Analysis preregistration	
Study was not preregistered	—
Other	
Data dictionary/analysis	Rácz-Brunner and Prevodnik (2025)

## Data Availability

Data can be accessed through the Open Science Framework (Rácz-Brunner & Prevodnik, 2025)
